# Uncertain choices with asymmetric information: how clear evidence and ambiguity interact?

**DOI:** 10.3389/fpsyg.2024.1509320

**Published:** 2024-12-19

**Authors:** Amir Hossein Tehrani-Safa, Atiye Sarabi-Jamab, Abdol-Hossein Vahabie, Babak Nadjar Araabi

**Affiliations:** ^1^Control and Intelligent Processing Centre of Excellence, School of Electrical and Computer Engineering, College of Engineering, University of Tehran, Tehran, Iran; ^2^School of Cognitive Sciences, Institute for Research in Fundamental Sciences (IPM), Tehran, Iran; ^3^Faculty of Governance, University of Tehran, Tehran, Iran; ^4^Cognitive Systems Laboratory, Control and Intelligent Processing Center of Excellence (CIPCE), School of Electrical and Computer Engineering, College of Engineering, University of Tehran, Tehran, Iran; ^5^Department of Psychology, Faculty of Psychology and Education, University of Tehran, Tehran, Iran

**Keywords:** ambiguity, information, decision-making, asymmetry, risk

## Abstract

Real-world decisions often involve partial ambiguity, where the complete picture of potential risks is unclear. In such situations, individuals must make choices by balancing the value of available information against the uncertainty of unknown risks. Our study investigates this challenge by examining how people navigate the trade-off between the favorability of limited evidence and the degree of ambiguity when making decisions under partial ambiguity. Participants (*n* = 77) engaged in a task where the level of ambiguity (small, medium, and large) and the favorability of the evidence (asymmetrically positive, neutral, and asymmetrically negative) were manipulated in a 3 × 3 design. We measured their attitude of ambiguity in each condition. The key finding reveals a bias in how participants perceived the unknown. They reacted to the unknown differently depending on the initial clues, filling in the missing information in a way that contradicted the evidence. When faced with positive evidence, participants were less tolerant of ambiguity than negative evidence. This means people were more careful when they received good news but less cautious when they received bad news. This bias was particularly pronounced when the ambiguity was low.

## Introduction

When making decisions under uncertainty, knowing the probabilities of different outcomes simplifies thinking about how people may approach choice problems by allowing us to apply the principles of rational decision theory (Kahneman and Tversky, 1979). This family of theories gives us clear guidelines about how one should decide, enabling straightforward hypotheses for what goes on in the decision-maker’s mind.

Under ambiguity, however, decision-makers cannot calculate risk. This introduces important difficulties in understanding how people make decisions with incomplete information, which incidentally happens to be the case with most everyday life decisions. As an example toy model, take Ellsberg’s famous demonstration: in a one-shot gamble to choose between a risky urn of 50 red (good) and 50 blue (bad) tokens and another ambiguous urn of 100 tokens with an unknown red/blue proportion, people tend to prefer choosing the former (risky) option over the latter (ambiguous) one (Ellsberg, 1961). Such “ambiguity aversion” may be interpreted to mean that individuals believe that the number of winning tokens in the ambiguous gamble must be fewer than in the risky one. This notion of subjective belief – called ambiguity attitude – about the likely structure of one’s ignorance could help us understand how the agent may fill out the missing information necessary to make a choice.

In many real-life situations, ambiguity does not necessarily become the complete absence of all information, but it can also indicate partially missing information. In such cases, one has to make up one’s mind with whatever partial information one has. “Partial” ambiguity attitude has been recently studied (Hsu et al., 2005; Huettel et al., 2006; Levy et al., 2010) by manipulating the relative size of the ambiguity while keeping the valence of the information neutral. Ambiguity aversion is also observed in the face of partial ambiguity. Examining ambiguity aversion under partial ambiguity raises important and new questions.

Available information often has some valence, sometimes promising benefit and other times cautioning against loss, pushing us toward or away from embracing the ambiguity vs. risk. In one study (Peysakhovich and Karmarkar, 2016) employing theoretical methods and behavioral experiments, asymmetric effects of positive and negative news were found. When available information supported a favorable outcome, ambiguity tolerance increased. However, unfavorable information did not affect the ambiguous attitude. Similar asymmetric treatments of positive and negative cues for decision-making under risk have been widely interpreted as the underlying cognitive basis of optimism bias (Lefebvre et al., 2017; Sharot, 2011).

**Figure 1:**
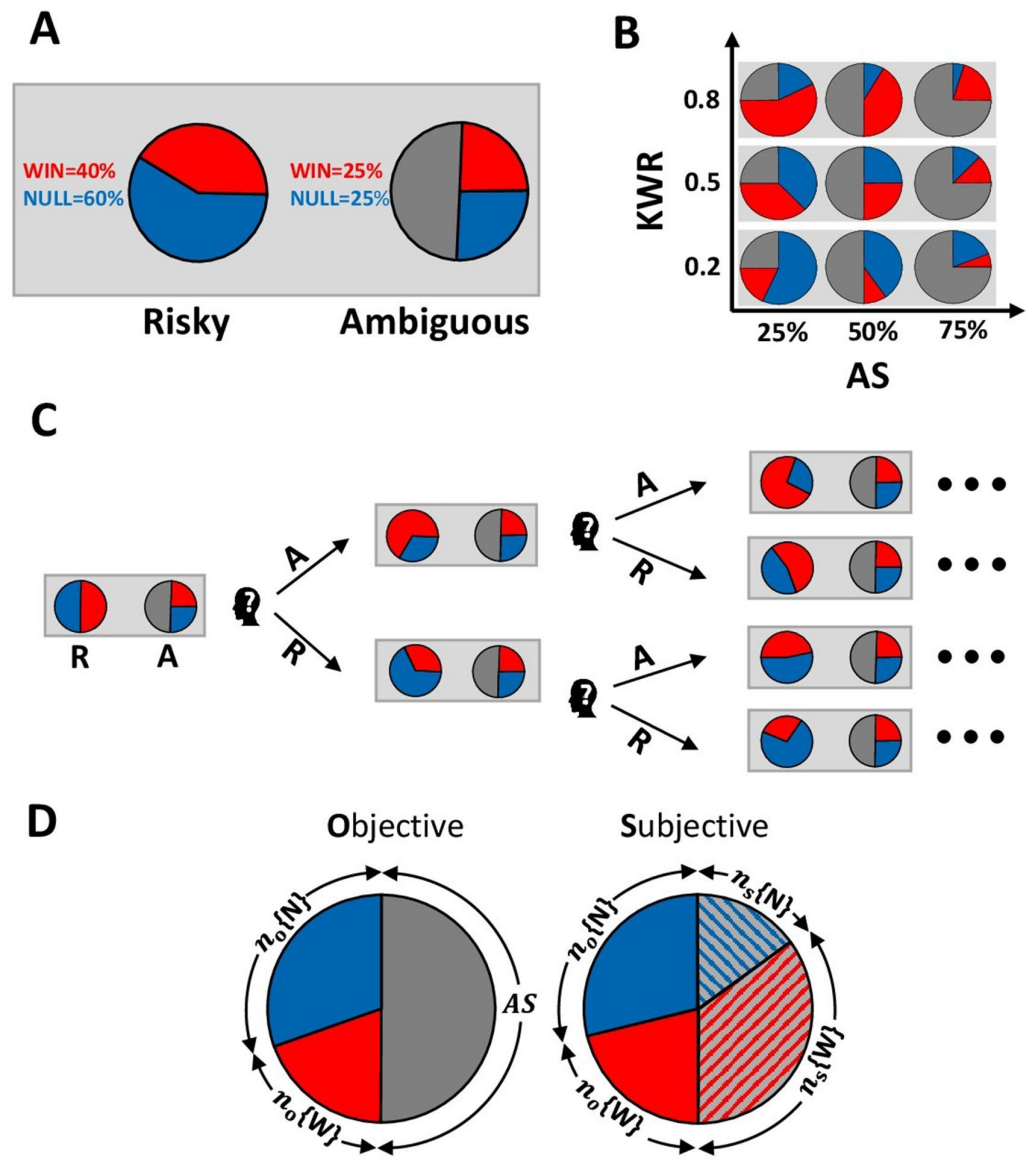
Stimuli and experimental design. **(A)** The pie chart on the left side represented the risky gamble, and the one on the right represented the ambiguous gamble. The proportion of tokens in known parts of both gambles was shown numerically next to the corresponding pie chart. **(B)** All ambiguous gambles were designed for the experiment. On the horizontal axis, the size of the hidden part increases from left to right. On the vertical axis, the number of winning tokens in the known part increases from down to up. KWR, known winning ratio; AS, ambiguity size. **(C)** Tree representation of staircase for one of the ambiguous gambles in one session. The winning ratio of the risky gamble was changed in the next block of the experiment depending on the participant’s choice. Choosing the risky (ambiguous) gamble staircase increases the proportion of null (winning) tokens in a risky urn on the next trial. A, ambiguous gamble; R, risky gamble. **(D)** Example of fractionation of ambiguous gamble by the participant. *n_o_*{.} indicates the number of tokens in the known part of the ambiguous gamble, and *n_s_*{.} relates to the subjective fractionation of the ambiguous part. AS, ambiguity size; W, wining tokens; N, null tokens.

By employing an experimental paradigm that combined risky and ambiguous decision-making, we examined how subjective probability may be constructed from positive vs. negative partial information as the participants chose between a risky option and another partially ambiguous option. We quantified ambiguity attitude in humans by comparing preferences between varying risky and partially ambiguous gambles. Each trial of our experiment presented a choice between playing a risky or a partially ambiguous gamble (Figure 1A) with the same payoff size. We systematically and orthogonally manipulated (Figure 1B) the proportion of ambiguity/information and the valence of information by changing the proportion of good/bad news (i.e., positive vs. zero rewards). By applying a staircase method (Figure 1C) borrowed from sensory psychophysics, we estimated the risky equivalent of each partially ambiguous gamble. This equivalent risky gamble allowed us to infer each participant’s subjective fractionation of ambiguity (Figure 1D).

Following the earlier works on optimism bias under risk (Sharot, 2011; Sharot and Garrett, 2016), we predicted that greater ambiguity tolerance should be observed when available information has positive vs. negative valence. Our results, however, demonstrated a much more nuanced behavior indicative of a flexible form of skepticism: when ambiguity size was tractable, subjective belief was sensitive to the valence of information; if the information was promising, ambiguity aversion increased, skeptically balancing the promising prospects of available evidence against the hazards of what might be hidden from the view. Conversely, when the information was disappointing, ambiguity tolerance increased, cautiously encouraging the participant to be more adventurous than what the available information guaranteed. When ambiguity was large, ambiguity attitudes were not affected by the valence of information.

## Methods

### Participants

A total of 77 healthy participants (mean age = 27.4, SD = 4.3) were recruited in the study, consisting of 36 females (19–37 years old) and 41 males (20–35 years old). Participants were from a wide range of academic disciplines, either at the graduate level or in the last semester of their first degree. Participants received monetary payment based on their decisions at the end of the experiment (see monetary payment).

### Ethics statement

All participants signed an informed written consent. The research was approved by Human Research Ethics Committee of the University of Tehran.

### Procedures

Participants were individually assessed for attitudes toward ambiguity. Each participant was briefed about the “game” and payment scheme. Participants knew that there was no “right answer” to any of the choices that they would face, and they were only required to report their preferences. They were informed that some of their decisions would be randomly used to calculate their monetary reward. Hence, their choices would not result in any loss. Each participant had played a training session before the experiment to become familiar with the task procedures.

### Main task

Our experiment consisted of 270 two-alternative forced-choice (2-AFC) trials that presented a choice between playing a risky or an ambiguous gamble with the same payoff. Two gambles were presented simultaneously on the computer screen as pie charts, which indicated the number of different tokens in each virtual urn, and both urns contained 100 tokens (Figure 1A). The red and blue areas of the pie charts represent the ratio of red and blue tokens. Participants were told that they would “win” if a red token was drawn from their chosen virtual urn. The known proportion of tokens was also shown numerically. Pie charts were rotated randomly to avoid using visual alignment in decisions between gambles.

To introduce ambiguity, a portion of one pie chart was blocked by gray. Participants were informed that each ambiguous gamble had an underlying winning ratio assigned to it, which was hidden from the participant in the gray section. In this way, calculating the expected value of the ambiguous gamble was impossible. Participants were assured that *a priori* winning ratios were fixed during the experiment and would not be changed by experimenters.

We systematically varied the properties of ambiguous gambles across trials (Figure 1B). We crossed three Ambiguity Sizes, AS (25, 50, and 75%), with three ratios of winning tokens over a total number of tokens in the known part, which we call the Known Winning Ratio, KWR (0.2, 0.5, and 0.8). Different values of KWR imply different probabilities of winning for participants. For example, KWR = 0.2 specified that in the known part of the urn, 20% of tokens were winning tokens, and 80% were null, so the delivered information is asymmetric in favor of losing the gamble. Following the same rationale, KWR = 0.5 implies an equal probability of winning vs. not winning. Finally, KWR = 0.8 meant that the information available to the participant favored winning.

The experiment was conducted in 3 sessions with 30 blocks of 3 trials. In each session, a fixed KWR was employed. Three ambiguous gambles of different ambiguity sizes were proposed within each block in randomly interleaved order. The order of which KWR to display in which session was randomized across participants. Participants had unlimited time to respond and did not receive feedback on the trials.

To estimate the equivalent risky gamble corresponding to each ambiguous gamble, we employed an approach similar to the staircase method with variable step size, which is commonly used in psychophysics studies (García-Pérez, 2011). The winning ratio of the risky gamble was adjusted adaptively across the session by a stochastic approximation staircase (Faes et al., 2007). If the participant preferred the risky gamble over the ambiguous one, then the winning ratio of the risky gamble was decreased in the next corresponding trial; if he/she preferred the ambiguous gamble, then the winning ratio of the risky gamble was increased. The changes in the winning ratio of the risky gamble were restricted to the AS. Thus, as the winning ratio of the risky gamble was changed depending on the participant’s choice, the subjective fractionation of the ambiguous part was estimated as the staircase covered the Ambiguity Size.

The staircase started with proposing the winning ratio of risky gamble equal to the number of winning tokens in the ambiguous gamble plus half of the AS. The initial step size of the staircase was equal to a third of AS and decreased as the participant reversed his/her choice. Decrement of the step size followed a harmonic series (i.e., AS/4, AS/5, …, AS/10) and remained constant when it reached AS/10 (Figure 1C). The choice of a large initial step size and its progressive decrement guaranteed the convergence of the staircase to the Point of Subjective Ambivalence (PSA) (Scheier et al., 1994). The minimum winning ratio proposed by the staircase was equal to the number of winning tokens in the known part of the ambiguous gamble, while the maximum winning ratio proposed to participants was given by fractioning all of the ambiguous parts as winning tokens.

After the experiment, the participants completed the Revised Life Orientation Test (LOT-R) (Holt and Laury, 2002). We used this questionnaire to measure trait optimism/pessimism, and its results did not affect the participants’ payment.

### Monetary payment

People might not perform realistically in hypothetical situations (Dimmock et al., 2016). Hence, we informed participants that we would randomly select one trial from each session (3 trials in total) to run the selected gamble in that trial for their monetary payment at the end of the experiment. We labeled numbers from 1 to 100 with red/blue colors with respect to the proportion of tokens of the chosen gamble in that trial. We asked participants to pick a number between 1 and 100. If the color assigned to the number was “red,” we paid them 100 K Rials (equivalent to $3). Each participant also received 100 K rials for participating in the experiment.

### Simulating agents to compare the experimental behavior with a number of possible alternative strategies

We anticipated that there might be a range of strategies to explain the ambiguity-resolving behavior. We simulated 1,000 agents for each of our suggested strategies (see details of cognitive strategies in the result section). The probability that the agent chooses an ambiguous gamble is calculated by a single logistic function: *P_Choice_* = *e*^*β.p*2^*/*(*e*^*β.p*1^ *+ e*^*β.p*2^), where *p*_1_ is the probability of winning in a risky gamble, and *p*_2_ is the subjective probability of winning in an ambiguous gamble. *β* is the slope of the logistic function or a noise parameter. For *β* near zero, choosing the ambiguous gamble or risky gamble has nearly the same probability. The probability of choosing the ambiguous gamble for high noise parameters tends to be 1. We used *β* = 0.2 for simulating random selection. Based on staircase results, we derived the corresponding AA in all nine experimental conditions for each simulated agent.

#### Task design

The risky gamble consisted of winning (red) and null (blue) tokens, where winning resulted in a 100 K rials payoff. The ambiguous gamble was similar to the risky gamble, but a portion of tokens was not disclosed to participants, and they did not know the ratio of winning and null tokens in this unclosed proportion. Gambles were not played until the end of the experiment. Participants reported their preference between ambiguous and risky gambles at each trial. No feedback was given about gambling outcomes during the experiment. Figure 1A depicts a sample trial consisting of a risky gamble (left pie chart) with fully known probabilities of outcomes: a 40% chance of winning and a 60% chance of getting nothing. The right pie chart depicts a symmetric ambiguous gamble with partially known probabilities of outcomes (25% < chance of winning <75%).

The winning ratio of the risky gamble varied systematically across trials to determine how the Known Winning Ratio (KWR) and Ambiguity Size (AS) influenced the participant’s choice. AS is the fraction of the ambiguous gamble covered by the gray sector. The experimental design combined three levels for AS [Ambiguity Size: small (25%), medium (50%), large (75%)], with three values for KWR [Known Winning Ratios: negative valence of information (0.2), neutral (0.5), positive valence of information (0.8)], giving rise to nine conditions. Figure 1B shows the nine conditions resulting from the 3 × 3 design.

Subjective attitudes toward ambiguity were elicited using a staircase technique with variable step size. For example, a run of the staircase for a designated ambiguous gamble (A) is shown in Figure 1C. Every time the participant chooses the risky gamble (R), the staircase proposes a risky gamble with an increased number of null tokens in the next step. Every time the participant chooses the ambiguous gamble, the staircase updates the risky gamble with an increased number of winning tokens.

#### Ambiguity attitude (AA)

We defined the Point of Subjective Ambivalence (PSA) between ambiguous and risky gambles as the average of the last 15 risky winning ratios proposed to the participant within a run of 30 trials. We used the PSA to infer how the participant must have fractionated the ambiguity into win (*n_s_*{*W*}) and null (*n_s_*{*N*}) subcomponents (Figure 1D). We then calculated the Ambiguity Attitude (AA) for each participant in each condition as shown in Equation 1:


(1) $$\mathrm{AA}=\frac{n_{s}\left\{W\right\}}{n_{s}\left\{W\right\}+n_{s}\left\{N\right\}}$$

AA is a number between 0 and 1. A value of 0.5 shows that the participant split the ambiguous part equally between winning and null tokens (Ambiguity Neutrality). Values higher than 0.5 indicate that the participant divided the ambiguity in favor of the winning tokens (Ambiguity Seeking, Figure 1D right pie chart). Values lower than 0.5 show that the participant interpreted the ambiguity negatively, favoring null tokens (Ambiguity Aversion).

## Results

**Figure 2:**
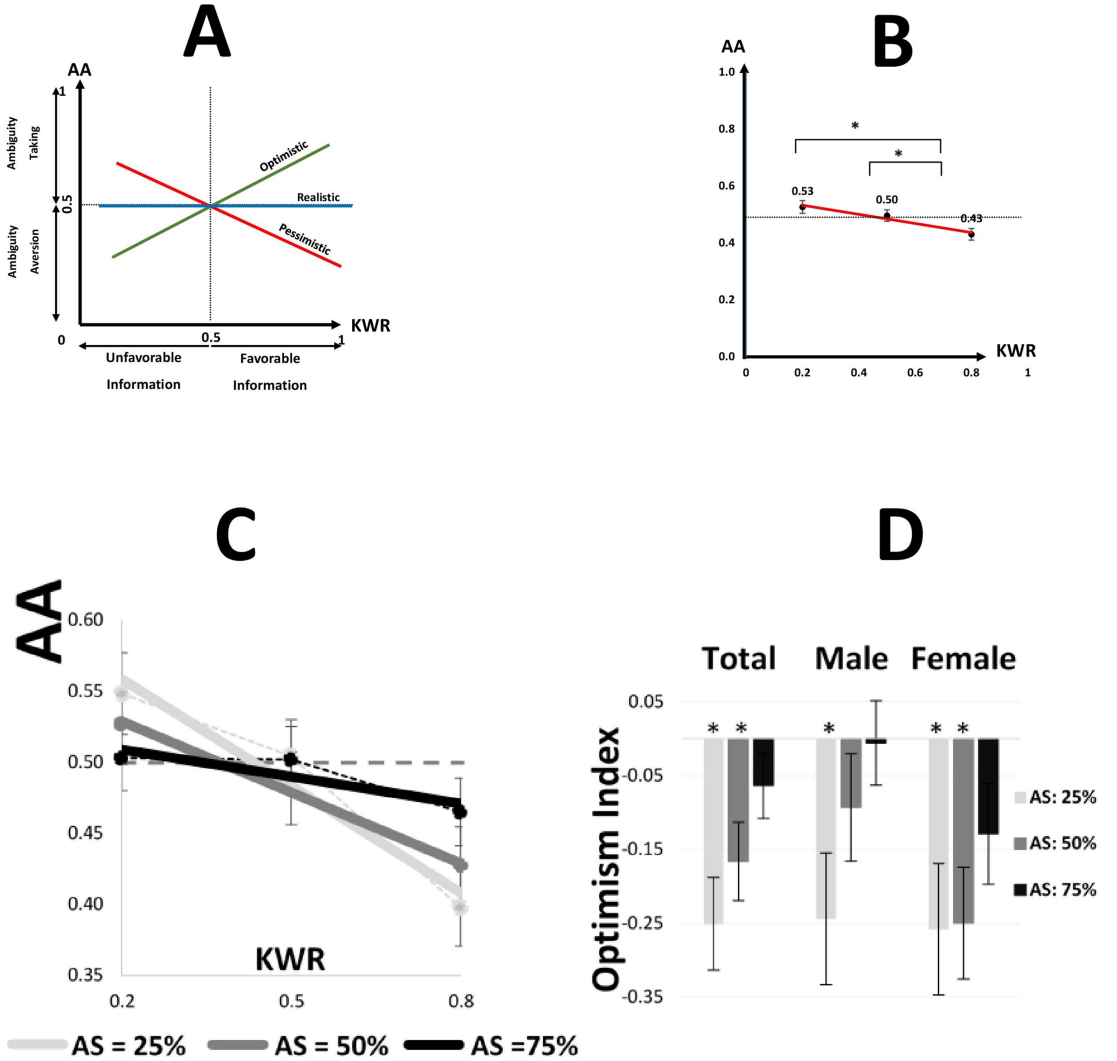
Ambiguity attitude and optimism Index in different conditions. **(A)** Schematic representation of the valence-dependent modifying account for our experiment. For an optimistic person, the higher KWR results in a higher ambiguity attitude (positive slope). A pessimistic person has a negative slope. A realist keeps the same AA irrespective of the valence of information (zero slope). **(B)** For a single participant, we calculated the average of AAs at a fixed KWR. The mean and SEM of averaged values have been illustrated. Pooled over all participants. * defines the significant difference of mean in a two-sample paired *t*-test (*p* < 0.05). AA, ambiguity attitude; KWR, known winning ratio. **(C)** A regression line was fitted for each participant. Then, the mean of regression lines is illustrated for each fixed AS pooled over the total dataset. **(D)** The mean and SEM of Optimism Indexes illustrated for each AS and gender, * defines *p* < 0.05.

By employing various KWRs (Figure 1B), we offered negative/neutral/positive valence of the information to the participants to measure the effect of the valence of the information on ambiguity attitude. Previously, the ambiguity attitude has been studied only under neutral information, where the probability of winning and not winning represented by partial information was equal (Hsu et al., 2005; Huettel et al., 2006; Levy et al., 2010). Our work goes beyond those previous studies by introducing different valences of information. We tested our hypothesis about the impact of the valence of information on the relative likelihood that participants attach to the ambiguous part. We predicted that the ambiguity attitude would be greater in positive than negative valence of information (Figure 2A).

A 3-way mixed ANOVA (KWR: negative, neutral, and positive; AS: small, medium, and large; gender: male and female; AA: dependent variable) was employed, showing a significant main effect of KWR [*F*(2,679) = 12.18, *p* = 6.3e-6]. There was also a significant effect of gender [*F*(1,679) = 7.24, *p* = 0.01], but no main effect of ambiguity size [*F*(2,679) = 0.14, *p* = 0.87] and no significant interaction between the independent variables (Supplementary Table S1).

The main effect of KWR on AA revealed a marked asymmetry in resolving ambiguity in different conditions. We calculated the average of AAs at a fixed KWR for each participant. A comparison between AA in positive vs. negative conditions (KWR = 0.2 vs. 0.8) confirmed that the AA in positive conditions was significantly less than the AA in negative conditions [paired *t*-test; *t*(76) = 3.43, *p* = 0.001] (Figure 2B). People were less ambiguity-tolerant in positive conditions relative to negative conditions. The ambiguity tolerance decreased as the information was more favorable. We concluded that dividing the structure of ignorance was biased with respect to the given evidence. People assume that the structure of the hidden part would be different from the structure of available evidence and fill out the missing bit of information differently when dealing with ambiguity. This kind of pessimism about given information in the domain of ambiguity needs more explanation and analysis.

Additional analysis showed that, on average, female participants were more ambiguity-averse than males (Supplementary Figure S1 and Supplementary Table S1). The lack of a main effect of AS on AA indicated that the size of ambiguity had not affected the Ambiguity Attitude. This was consistent with previous studies on decision-making under partial ambiguity (Inukai and Takahashi, 2009; Lopez-Paniagua and Seger, 2013).

To explain how the subjective structure of probability distribution in the hidden part was biased with the accessible information in the known part, we defined the Optimism Index (OI) by the following Equation 2:


(2) $$OI\;\left(\mathrm{Optimism}\;\mathrm{Index}\right)=\frac{\Delta AA}{\mathrm{\Delta KWR}}=\frac{AA_{KWR_{2}}-AA_{KWR_{1}}}{KWR_{2}-KWR_{1}}$$

Let us explain how the novel optimism index works. An optimistic person has a positive optimism index. This means that she has more ambiguity tolerance (less ambiguity aversion) in favorable conditions than in unfavorable conditions. Therefore, for an optimistic person, the higher KWR results in a higher ambiguity attitude (positive optimism index). Inversely, a pessimistic person has a negative Optimism Index. Her ambiguity aversion in the positive condition is bigger than in the negative condition. If the information is biased toward the winning, she assumes fewer winning tokens in the hidden part. A realistic person does not change her ambiguous attitude due to the valence of information. In other words, the proposed information cannot change her subjective belief about the distribution of tokens in the hidden part (Figure 2A and Supplementary Figure S2).

The novel introduced the Optimism Index, which measures people’s sensitivity to the given information. We know that an ambiguous attitude also traces some optimism/pessimism trait. However, we should emphasize that the Optimism Index measures different issues. When we call someone ambiguity averse, she generally dislikes ambiguous options and perceives ambiguity as undesirable. However, here, the Optimism Index measures how she changes her subjective probability in line with accessible data. For example, if the biased structure to winning could lead to reduced ambiguity aversion. From this definition, we understand that someone could have a positive optimism index, and she could also be ambiguity-averse.

To calculate the OI, we regressed AA on KWR values for each AS for each participant. Figure 2C shows the regression line for each Ambiguity Size pooled across all participants. Figure 2D shows the optimism index for each level of AS separately for male and female participants and the entire dataset. In our empirical data, a two-way ANOVA (dependent variable: Optimism Index) with factors of AS and gender showed that there was no main effect of gender [*F*(1,225) = 2.47, *p* = 0.12] but a marginally significant main effect of AS [*F*(2,225) = 2.92, *p* = 0.056] on slopes. A comparison between OI in AS = 75% with zero confirmed no significant difference [one sample *t*-test with zero; AS = 75%; *t*(76) = −1.42, *p* = 0.16]. When the ambiguity size was large, people tended to be more realistic. Moreover, the OIs in small and medium ambiguity size conditions were significantly less than zero [one sample *t*-test with zero; AS = 50%: *t*(76) = −3.14, *p* = 0.0024; AS = 25%: *t*(76) = −3.99, *p* = 1.00E-04] (Supplementary Table S2). When the ambiguity size was tractable, people tended to be pessimists.

Additional control measures showed that optimism indexes were not correlated with participants’ trait optimism (LOT-R) (AS = 25%: *r* = 0.03, *p* = 0.81; AS = 50%: *r* = 0.1, *p* = 0.39; AS = 75%: *r* = 0.18, *p* = 0.13) (Supplementary Figure S3).

To develop a rigorous theoretical framework for decision-making under ambiguity with asymmetric information, we require a weighting distortion function that can accommodate both symmetric and asymmetric information scenarios. To identify this function, we adopted the approach of basing the distortion function on observed indifference between a risky gamble with a known probability of $\left(1-AS\right)*KWR+AS*\mathrm{AA}$ winning and an equivalent ambiguous gamble (AS, KWR).

We begin by introducing three well-established distortion functions from the literature that are applicable to our data. Subsequently, we present our proposed distortion function, which is inspired by one of these existing functions, and further inform you based on our empirical findings.

The first model is the inverse S-shaped distortion function, introduced by Abdellaoui et al. (2011):


(3) $$w={\left(\exp\left(-{\left(-\ln\left(P_{\mathrm{objective}}\right)\right)}^{\alpha }\right)\right)}^{\beta }$$

In Equation 3 *α* represents the index of insensitivity, and *β* represents the index of pessimism.

The second weighting function considers the effect of ambiguity in a linear manner (Gilboa and Schmeidler, 1989):


(4) $$w=P_{\mathrm{objective}}-\frac{AS}{2}*\gamma$$

In Equation 4, *γ*, the fitted ambiguity aversion parameter ranges from −1 to 1, with 1 indicating maximum aversion. This differs slightly from our definition of ambiguity attitude (AA), which ranges from 0 to 1, with 1 indicating maximum ambiguity seeking.

Finally, the third model, employed by Hsu et al. (2005), incorporates the effect of ambiguity through an exponential structure.


(5) $$w=P_{\mathrm{objective}}^{\left(1+\gamma \times AS\right)}$$

In Equation 5, *γ* is the parameter that measures ambiguity aversion.

We now turn to the development of our proposed model, which was informed by our empirical results. We constructed a generalized linear mixed-effects model with a group-level intercept, as shown in Equation 6, treating AS and KWR as independent variables and AA as the dependent variable.


(6) $$\mathrm{Initial}\;\mathrm{Linear}\;\mathrm{Model}:\mathrm{AA}=C_{0}+C_{1}\times AS+C_{2}\times KWR$$

Consistent with our previous 3-way ANOVA (Supplementary Table S1), linear model fitting revealed significant effects of the intercept and KWR (*p* < 0.001) but not AS (*p* = 0.74).

Therefore, we built a revised linear model with KWR as the sole independent variable and an intercept term:

(7) $$\text{Suggested linear model:}\mathrm{AA}=K_{0}+K_{1}\times KWR$$

Fitting this model to the entire dataset, we obtained a coefficient of −0.16 for KWR and an intercept of 0.56, both of which were statistically significant (*p*-value < 0.0001). The negative coefficient for KWR aligns with our behavioral findings (Figure 2C), demonstrating a negative relationship between ambiguity attitude (AA) and KWR. Furthermore, a Pearson correlation analysis yielded a correlation coefficient of *ρ* = −0.17 (*p* < 0.0001), confirming a significant negative correlation between AA and KWR.

The foregoing analysis adopted a fixed-effects framework for the entire dataset. To incorporate potential heterogeneity in the AA-KWR relationship across individuals, we estimated subject-specific models based on Equation 7, allowing for individual-level parameter variation.

Leveraging our established understanding of the equivalent chance of winning for an ambiguous gamble (AS, KWR), we can propose a weighting function with the following structure as shown in Equation 8:


(8) $$w=\left(1-AS\right)\times KWR+AS\times \mathrm{AA}$$

Furthermore, the objective probability is obtained by dividing the ambiguous part equally between the possible outcomes (Equation 9):


(9) $$P_{\mathrm{objective}}=\left(1-AS\right)*KWR+AS/2$$

So, we have:


(10) $$w_{\mathrm{model}}=P_{\mathrm{objective}}–\frac{AS}{2}+AS\times \mathrm{AA}$$

And finally, by replacing Equation 7 into Equation 10, we have Equation 11, as follows:


(11) $$w_{\mathrm{model}}=P_{\mathrm{objective}}–\frac{AS}{2}+AS\times \left(K_{0}+K_{1}\times KWR\right)$$

We define $w_{\mathrm{empirical}}$ as the probability of winning in the risky equivalent gamble for each ambiguous gamble (AS, KWR). Note that here, $w_{\mathrm{empirical}}$ is calculated from the behavioral data, with AA extracted from the subject’s behavior for each ambiguous gamble individually ( $w_{\mathrm{empirical}}=\left(1-AS\right)\times KWR+AS\times AA_{\mathrm{empirical}}$ ). We then fit $w_{\mathrm{empirical}}$ vector (9 conditions of the experiment) to the proposed w function to determine the best-fitting parameters for each subject separately.

To compare the performance of our proposed weighting function, we evaluated it against three prominent weighting functions commonly used in decision-making under ambiguity research. Having all models, we fitted the $w_{\mathrm{empirical}}$ vector to each weighting function for each subject individually.

We calculated the error for each model for each of the 77 subjects. Subsequently, we employed a one-way ANOVA test to compare the error values across the different models. Each column in the ANOVA analysis consisted of the error of each model for all 77 subjects. The results indicated a significant error difference between the models (*F* = 3.39, *p*-value = 0.018). A *post hoc t*-test comparing our model with the next best-performing model (inverse S-shaped distortion function [Equation 3]) revealed a significant difference between them (CI = [0.013, 0.025], SD = 0.02, *p*-value < 0.001, df = 76). Please refer to the Supplementary material for details on the model comparison. This file contains predictions of each fitted distortion function for selected subjects, a boxplot of the error distribution for the four competitive models, and a detailed report of the *t*-tests comparing our model to the other models.

**Figure 3:**
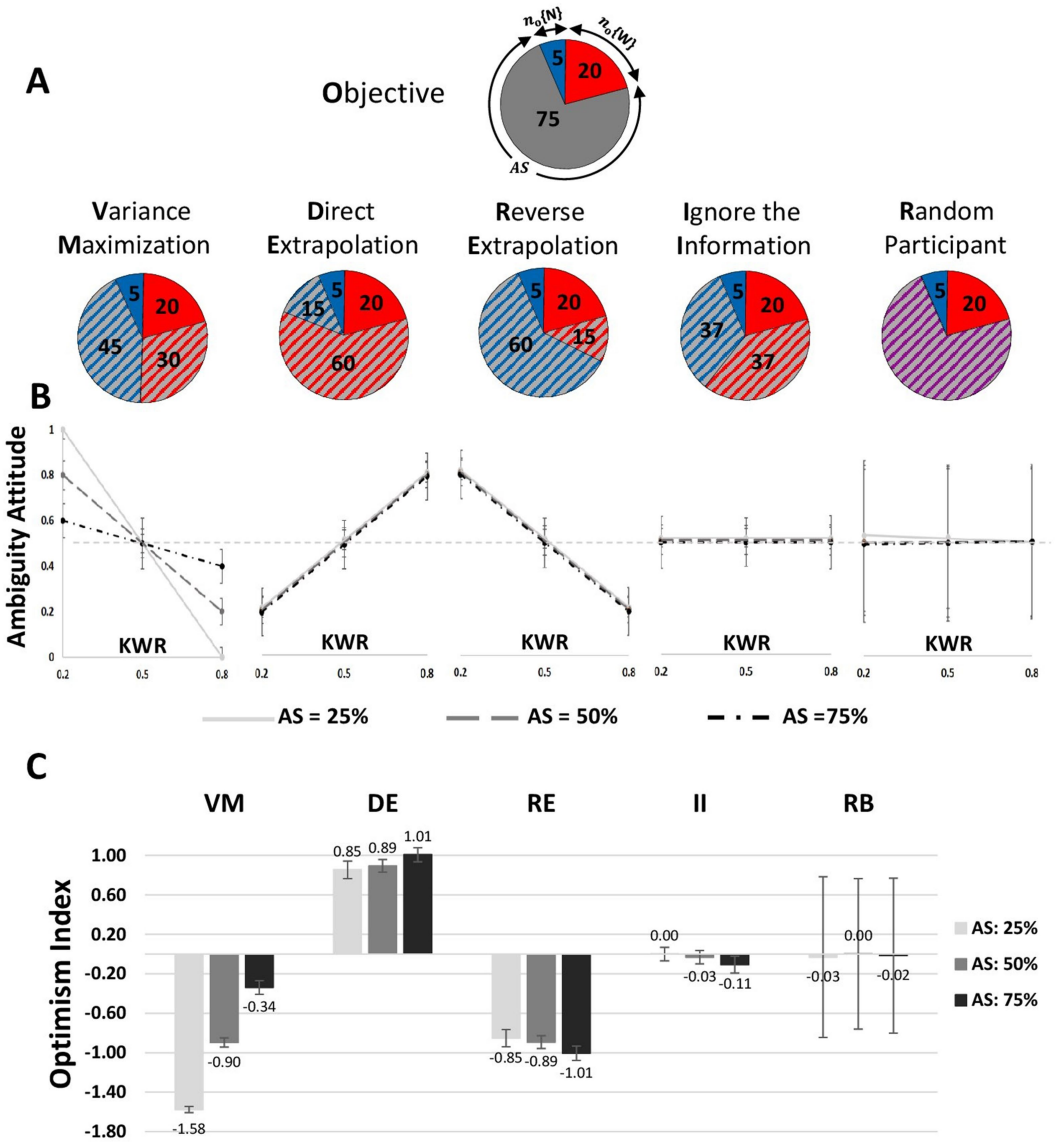
Different plausible strategies for resolving ambiguity and their model predictions are compared with empirical results. **(A)** Quantitative calculation of resolving ambiguity with examples for different strategies. *n_o_*{.} indicates the number of tokens in the known part of the ambiguous gamble, and *n_s_*{.} relates to subjective fractionation of the ambiguous part, AS, ambiguity size; W, wining tokens; N, null tokens. **(B)** Model predictions for each strategy. For all strategies, to estimate the optimism indexes, we simulated 1,000 agents for each strategy, and next, for each agent, we calculated values of AA. The mean and standard deviation of AA values have been illustrated. In these figures, the *X*-axis corresponds to three steps of KWR, and the *Y*-axis corresponds to computed ambiguity attitude (AA). Each line indicates the relationship between KWR and AA (optimism index) in a fixed AS. At KWR = 0.5 and with different ambiguity sizes, the values of AA are equal to 0.5 by following any of the considered strategies. **(C)** Mean and SEM of Optimism Indexes for alternative strategies illustrated for each AS.

In light of the proposed model, we can compare the empirical results to a number of plausible cognitively inspired hypotheses for the mental process shaping our subjects’ decision-making under ambiguity (Figure 3B). A key strength of this approach is that the predictions drawn from these seemingly similar hypotheses are radically different once they are applied to the context of the experimental setup. As a result, even a qualitative comparison of the data (Figures 2C,D) to the predictions (Figures 3B,C) communicates our point sufficiently.

### Cognitive strategies

#### Variance maximization

If the participants interpreted every ambiguous gamble as a 50–50 risky urn, then the subjective ambivalence for each of the nine conditions would indicate that (*n_S_*{*W*} + *n_O_*{*W*} = *n_S_*{*N*} + *n_O_*{*N*} = 50). This would be equivalent to assuming *maximum* var*iance* in the ambiguous gamble (Figure 3A). A participant following this extremely simple and intuitive strategy would compare the probability of winning in a risky gamble with 50%. Remarkably, such a simple strategy would correspond to a very elaborate pattern of different negative slopes for the relationship between KWR and AA for different ambiguity sizes (Figure 3B).

#### Direct extrapolation

Participants may fractionate the ambiguous part in the same proportion as the known part (Figure 3A). The choice would involve comparing the ratio of winning tokens in the known part with the ratio of winning tokens in the corresponding risky gamble, where the quantitative outcome is (*n_S_*{*W*}/*n_S_*{*N*} = *n_O_*{*W*}/*n_O_*{*N*}). This strategy predicts a unique positive slope for the relationship between AA and KWR (Figure 3B).

#### Reverse extrapolation

A paranoiac participant may assume that the portion of tokens in the ambiguous part is the inverse of the proportion displayed in the known sector (Figure 3A). The quantitative outcome is (*n_S_*{*W*}/*n_S_*{*N*} = *n_O_*{*N*}/*n_O_*{*W*}), which corresponds to predicting the same fixed negative slope for all ambiguity sizes (Figure 3B).

#### Ignoring the information

Participants may not incorporate the partial information to resolve ambiguity, always splitting the ambiguous part in half. Participants following this strategy would not adjust their belief in response to variation of partial information. AA would be independent of KWR (Figure 3B) but for a fixed intercept indicating “Ambiguity Aversion” and “Ambiguity Seeking.”

#### Random behavior

Finally, a useful null hypothesis is the one. These predictions were obtained by simulating the agent taking up each strategy (see Method) and calculating the simulated agent’s AA (Figure 3B).

Both Variance Maximization (VM) and Reverse Extrapolation (RE), as illustrated in Figure 3B, exhibit a decreasing relationship between AA and KWR. However, the VM weighting function remains constant at 0.5, independent of KWR and AS. In contrast, the observed variation of AA concerning KWR (Figure 2C) resembles the VM plot in Figure 3B, as suggested by visual inspection. Conversely, a formal analysis reveals a stronger alignment between the structure of our proposed weighting function and the RE cognitive strategy (see Supplementary material for AA of different cognitive strategies).

Comparing our proposed model with the RE strategy, we observe structural similarities, with the primary difference in the coefficients associated with AA. The coefficients of KWR in AA_RE_ are −1, which is more extreme, leading to significant variations between conditions. Conversely, the coefficient of AA in our fitted model is more moderate (−0.16), resulting in a smoother variation. Additionally, the intercept of AA in our fitted model is approximately 0.56, which is close to 0.5, suggesting a strategy where subjects may simply ignore the information and equally divide the unknown probability between winning and losing. From this perspective, we can interpret the subjects’ strategy as a tempered version of RE, where the variation of AA is centered and confined to a narrower range around 0.5, thus exhibiting similarities to the VM strategy in visual representation.

A further hypothesis drawn from the Variance Maximization strategy is that if the available information is already consistent with maximum variance (i.e., KWR = 0.50), the participant should have a much simpler task requiring much less cognitive effort to disambiguate the unavailable information. This would lead to the prediction that response times should be shorter when KWR = 0.50 compared to when KWR <> 0.50. On the other hand, many previous studies have shown that choice response times in human and non-human primates (Zylberberg et al., 2016; Kiani and Shadlen, 2009) increase with variance in the evidence. These previous studies would predict maximum response time in KWR = 0.50.

**Figure 4:**
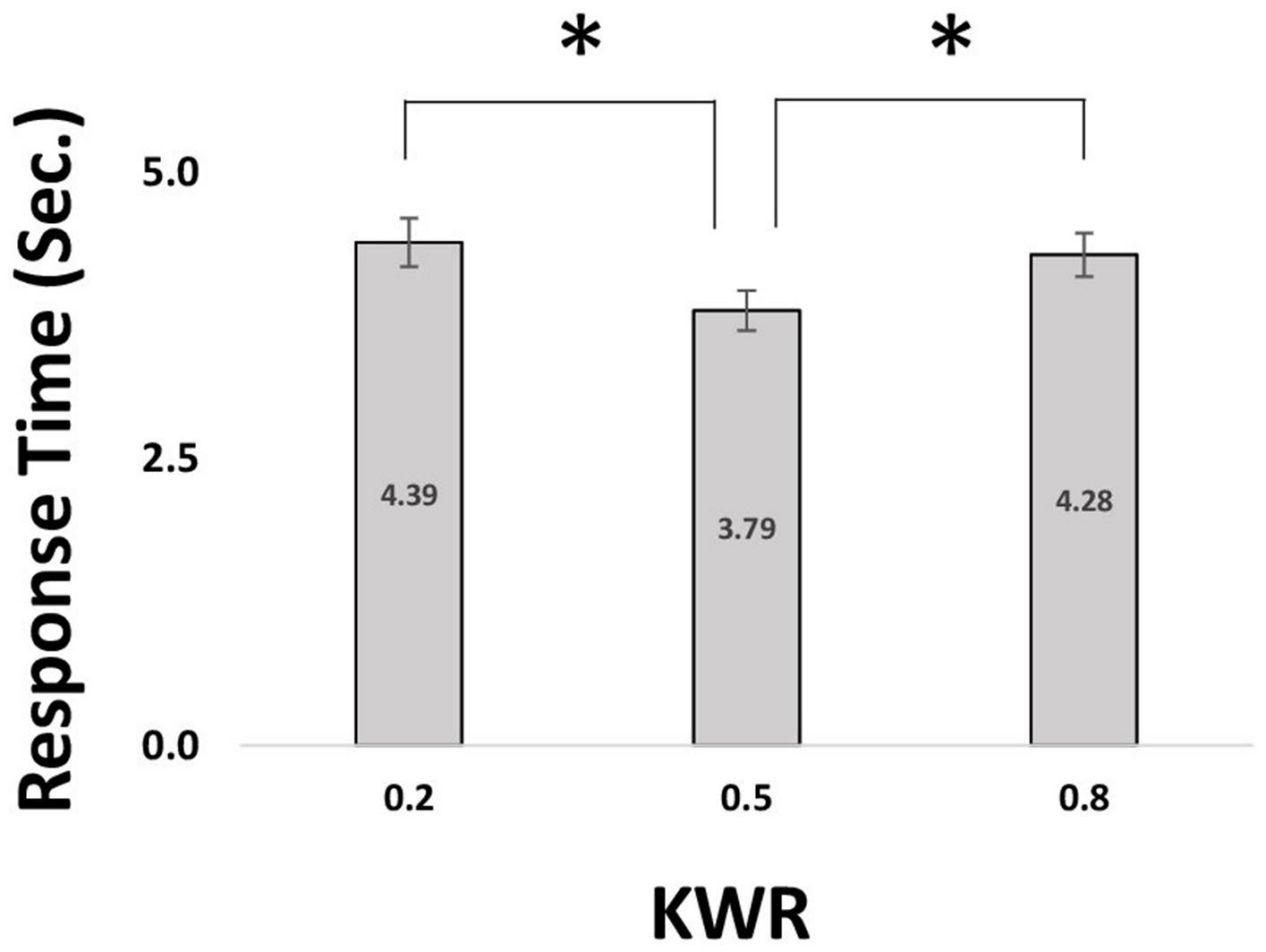
Comparing response time in different known winning ratio (KWR). For a single participant, we calculated the average values of RT in 3 different ASs at a fixed KWR. The mean and SEM of averaged values have been illustrated. * indicates a significant difference of means by two-sided paired *t*-test (*p* < 0.05). RT, response time; KWR, known winning ratio.

We analyzed the response times (RTs; Figure 4) of choices between risky and ambiguous gambles (RTs longer than 20 s were excluded from the analysis). A one-way ANOVA on RTs indicated that KWR has a main effect [*F*(2,76) = 2.73, *p* = 0.05]. There was a significant difference between conditions with negative/positive valence of information (KWR = 0.2 and 0.8) and the neutral condition (KWR = 0.5) [paired *t*-test; 20% vs. 50%; *t*(76) = 3.44, *p* = 0.0009; 80% vs. 50%; *t*(76) = −3.14, *p* = 0.002, Figure 4].

## Discussion

Not much is known about the role of information in constructing subjective belief under ambiguity, where the probability distribution over uncertain events is partially or completely unknown. To address this question, our study focused on how individuals use the evidence to disambiguate what they do not know.

We combined a staircase procedure commonly used in sensory psychophysics (García-Pérez, 2011; Treutwein, 1995) with a classical risky choice paradigm in behavioral economics to estimate and extract the participants’ ambiguous attitudes. We directly elicited participants’ ambiguous attitudes by revealing their preferences in choosing between risky and ambiguous gambles in the context of an adaptive staircase. To test our main hypothesis, we introduced a novel approach by employing partial ambiguity, which goes beyond previous studies of decision-making under ambiguity (Levy et al., 2010; Tymula et al., 2012).

Some recent studies investigate how information could change ambiguity aversion (Jia et al., 2020), they taught people about the Ellsberg paradox and their own potentially suboptimal decisions in ambiguity. Results showed that this intervention reduced participants’ ambiguity aversion. Generally, it showed that information could modify human suboptimal strategies in the face of ambiguity.

Peysakhovich and Karmarkar (2016) employed both empirical and theoretical methods to investigate how favorable and unfavorable information can influence the perceived value of ambiguous options.

They showed that information added in favor of the winning raises the value of ambiguous gambling in the eyes of gamblers. However, no effect was found when the information favored losing the gamble. Their valuable work was distinct from our work in some aspects. First, they measured willingness to pay (WTP), balancing two factors: ambiguity aversion and subjective likelihood estimates, while we only focused on ambiguity aversion and how it could be swung by asymmetrical partial information. Second, most of their conditions were special cases that were excluded from our task, for example, (0 and 25%) and (50 and 0%) in their task, which equals KWR = 0 and KWR = infinity, respectively.

Previous studies used a parametric computational model to interpret the ambiguity attitude. A softmax function has often been employed to model the probability of choosing the ambiguous gamble. Those previous works estimated the ambiguity attitude by applying a non-linear optimization constrained by the participant’s choice, which requires numerous assumptions about the shape of the distribution (Levy et al., 2010; Tymula et al., 2012). In our study, we fixed the monetary payment for both gambles and changed the winning ratio of risky gambles. Our non-parametric method based on the staircase procedure empowered us to directly measure the ambiguity attitude. Therefore, we do not make any assumptions that may have affected the computational analyses.

Our results also showed a valence-dependent asymmetry in how people handle promising and disappointing information to decide what they do not know. People do not fully trust the available evidence when they face ambiguity. Promising information pushes people to change their beliefs skeptically as they balance the promising prospects of available evidence against the hazards of what might be hidden. Conversely, disappointing information fails to thwart people from being adventurous about what might be hidden versus what the evidence suggests.

In an unknown environment, people might have interpreted the evidence as a deceptive effort, as if somebody might have wanted to lead them on to take a bad risk or lose some benefits (Shepperd et al., 2000; Sweeny and Krizan, 2013). Our results are consistent with these previous reports on context’s impact on valence’s role in the ambiguity domain.

Although there are a number of advantages to holding positive expectations, there seem to be obvious disadvantages to ignoring negative information, such as underestimating risks. The asymmetric belief formation has been blamed for a host of disasters, such as overly aggressive medical decisions (Paling, 2003), ill-preparedness in the face of natural catastrophes (Paton, 2003), and financial collapse (Shefrin, 2009). Moreover, positively biased views of the self can lead to error and cost, as shown, for example, in overconfident traders (Barber and Odean, 1999). In an unknown environment, such a pessimistic attitude could help us handle information better when deciding what we do not know.

A previous study concluded that individuals with greater ambiguity tolerance have a greater tendency to trust other people during social decisions (Vives and Feldmanhall, 2018). Although some previous studies in the non-social domain illustrated that individuals with higher ambiguity tolerance are more optimistic about the future according to the LOT-R test (Pulford, 2009), our new measure showed that the amount of optimism or pessimism about life is not related to the optimism in the ambiguity domain. Future studies will be needed to disentangle the relationship between personality and behavior in ambiguity.

## Data Availability

The datasets presented in this study can be found in online repositories. The names of the repository/repositories and accession number(s) can be found: https://osf.io/9y7uq/.
