# Supplementary material for: Uncertain choices with asymmetric information: how clear evidence and ambiguity interact?
Source: Front Psychol. 2024 Dec 19;15:1509320. doi: 10.3389/fpsyg.2024.1509320 (PMC11696535; doi:10.3389/fpsyg.2024.1509320)
Supplement: Supplementary file 1 [file Data_Sheet_1.DOCX]

**Figure S4: boxplot of the error distribution for the four competitive models**

**
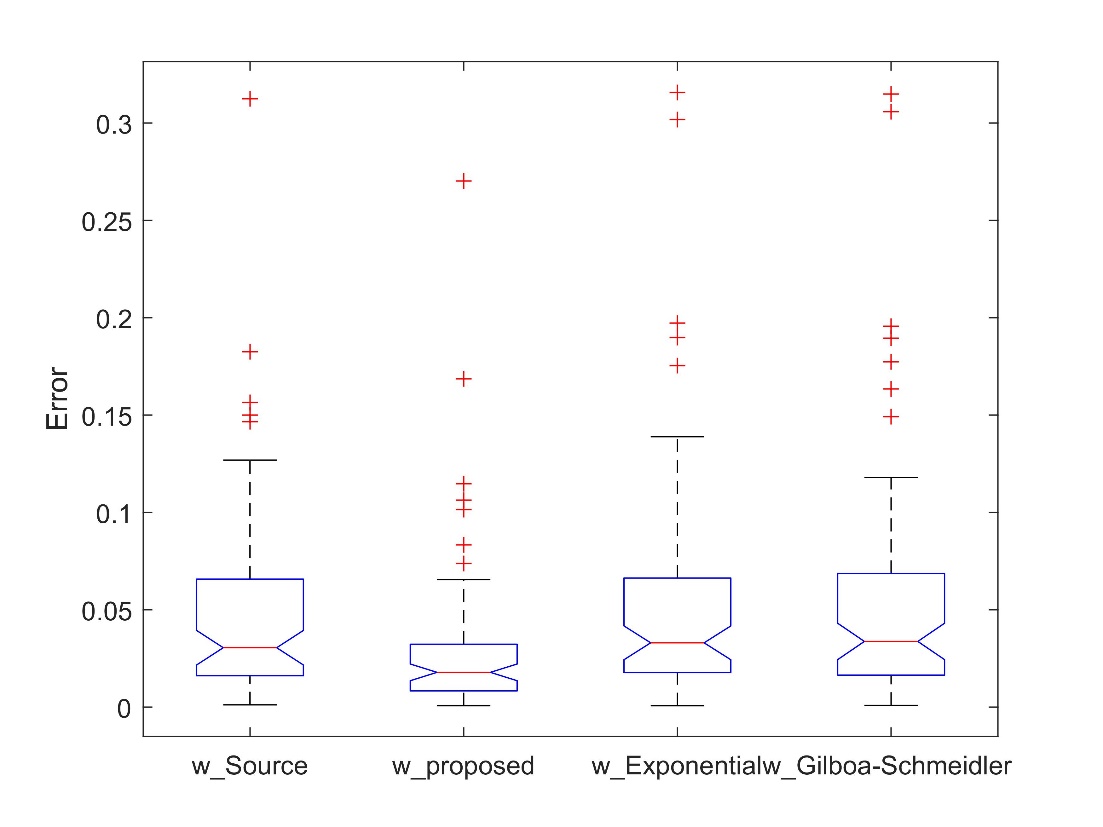
**

We conducted three post-hoc t-tests to compare our model to the other three models. The results indicate that the error of our model was significantly lower than that of the other models, as summarized in Table S3

**Table S3: a detailed report of the t-tests comparing our model to the other models**

|  | tstat | sd | pvalue | df | ci |
| --- | --- | --- | --- | --- | --- |
| Source Function | 6.33 | 0.02 | <0.001 | 76 | [0.013 0.025] |
| Gilboa-Schmeidler | 4.83 | 0.04 | <0.001 | 76 | [0.014 0.033] |
| ExponentialFunction | 4.97 | 0.04 | <0.001 | 76 | [0.015 0.034] |

**Figure S5: Predictions of each fitted distortion function for selected subjects**

| 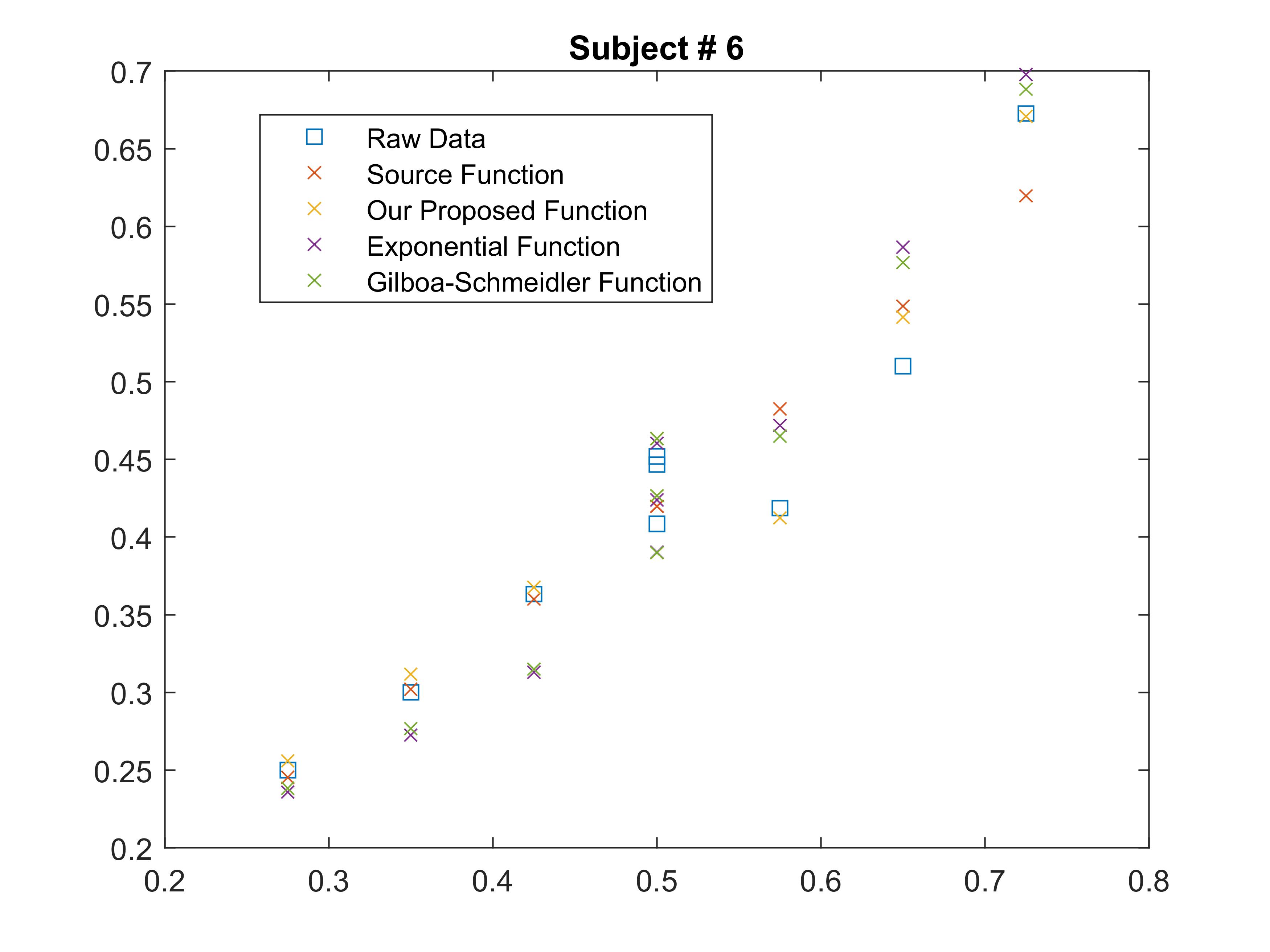 | 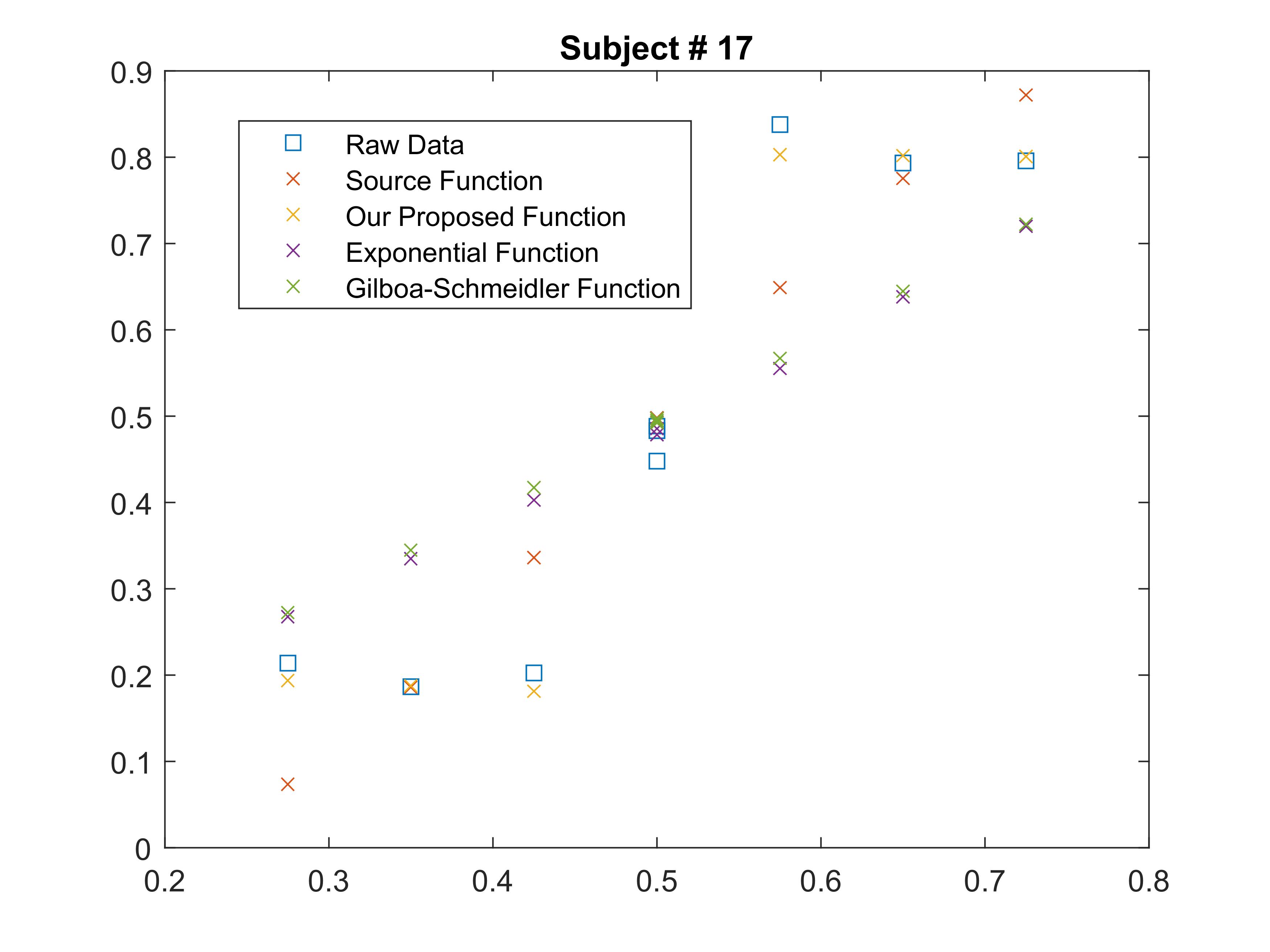 |
| --- | --- |
| 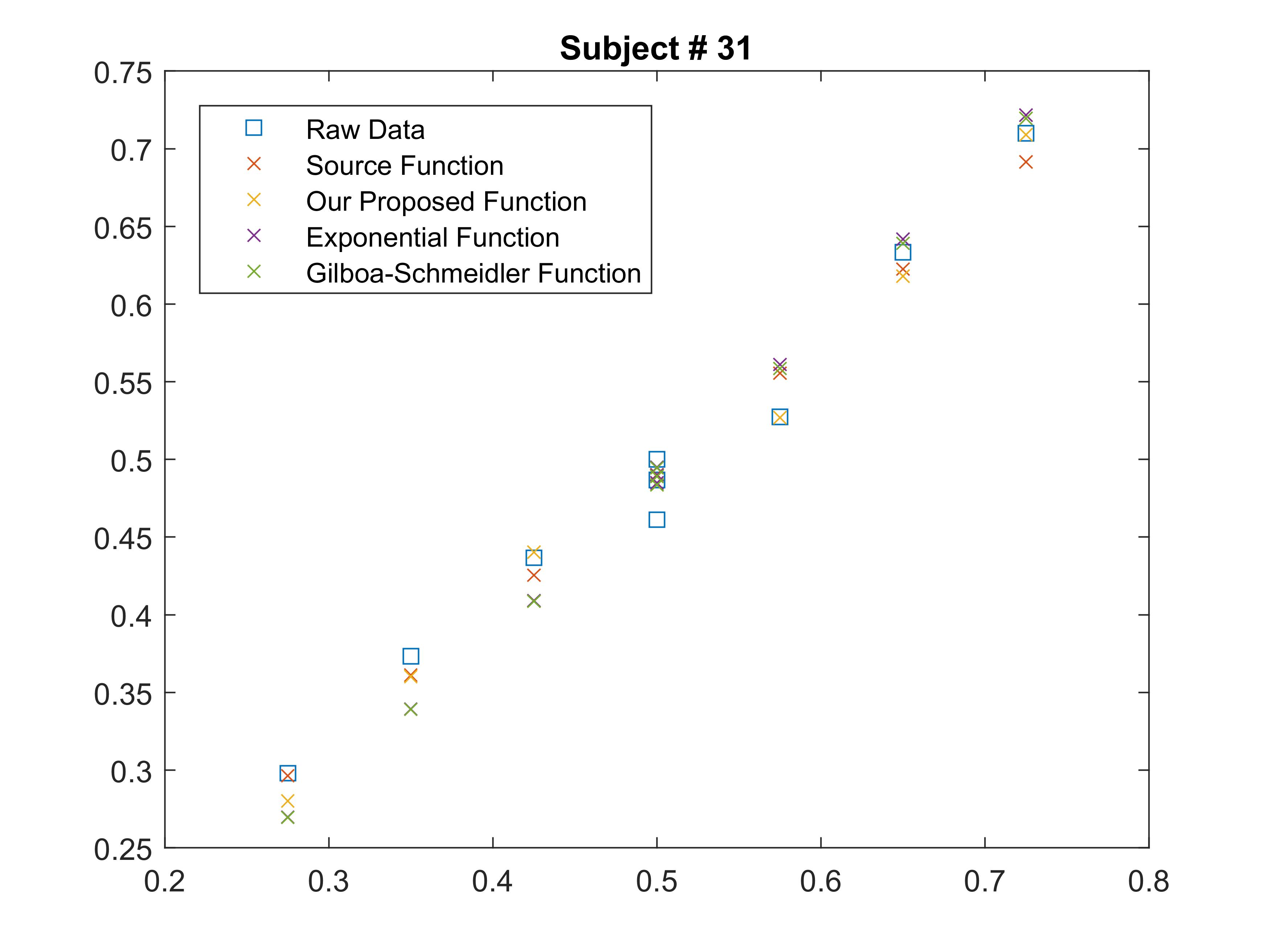 | 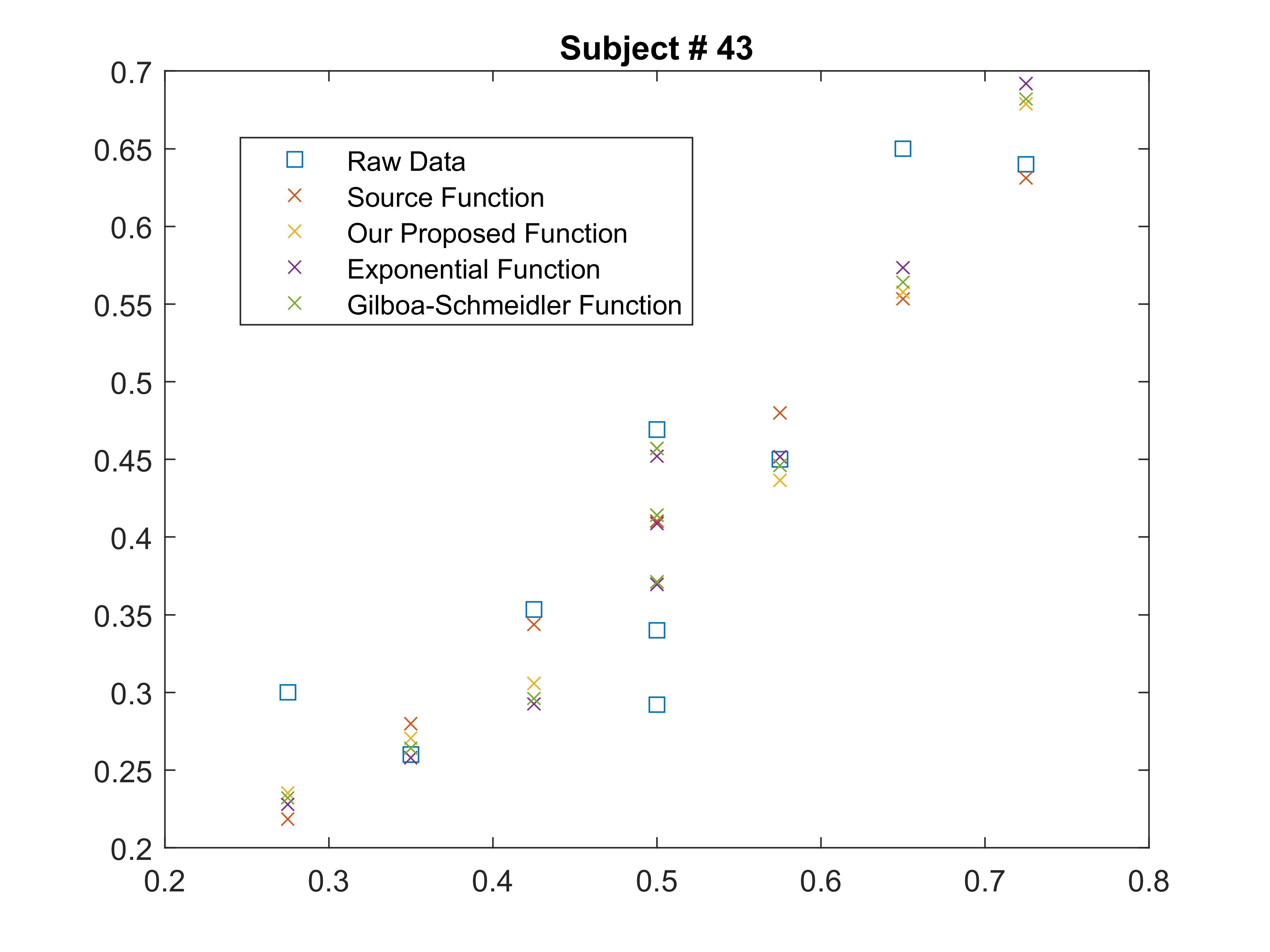 |

**Figure S6: Inverse S-shaped source function for the selected subjects**

| 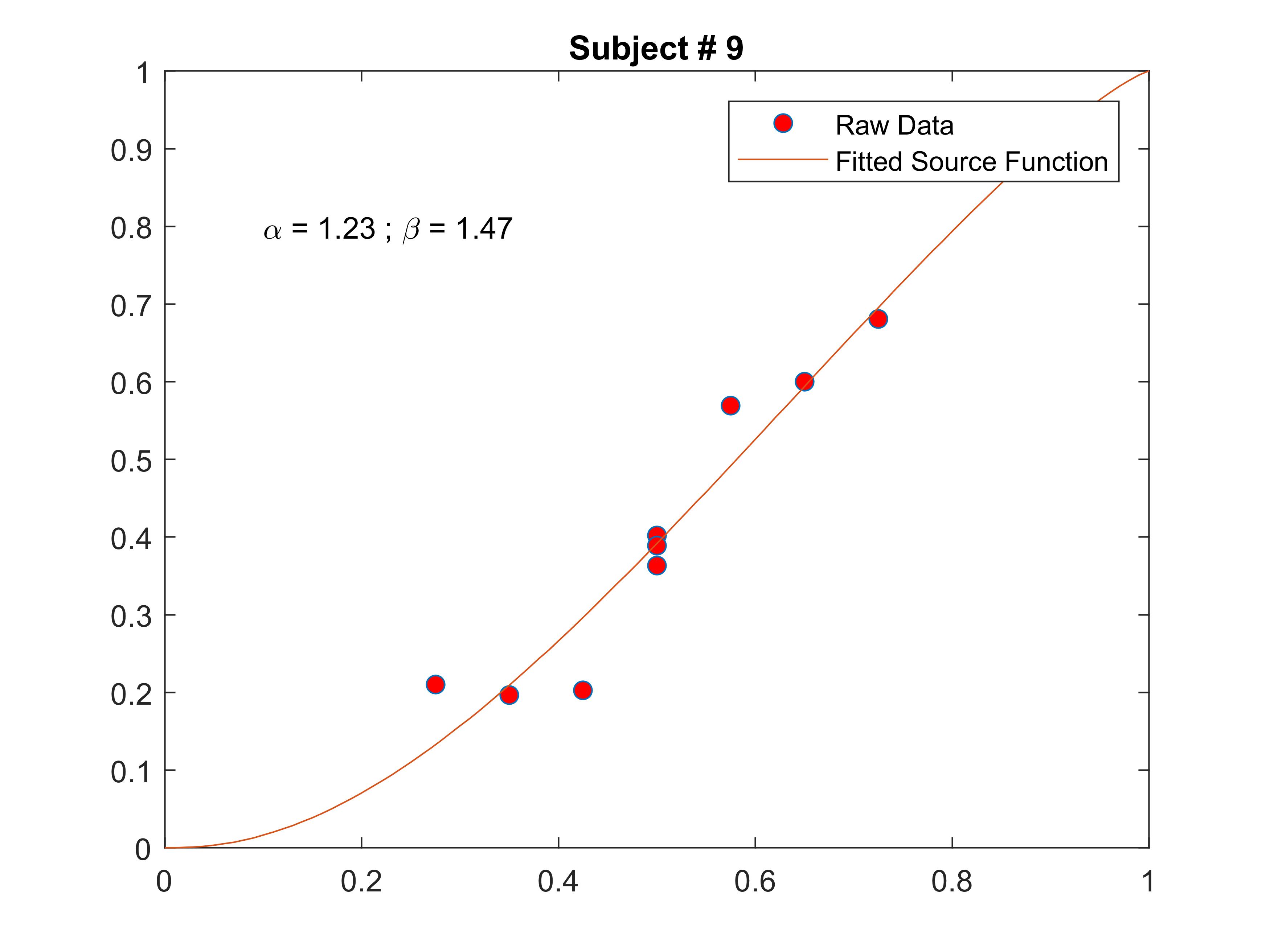 | 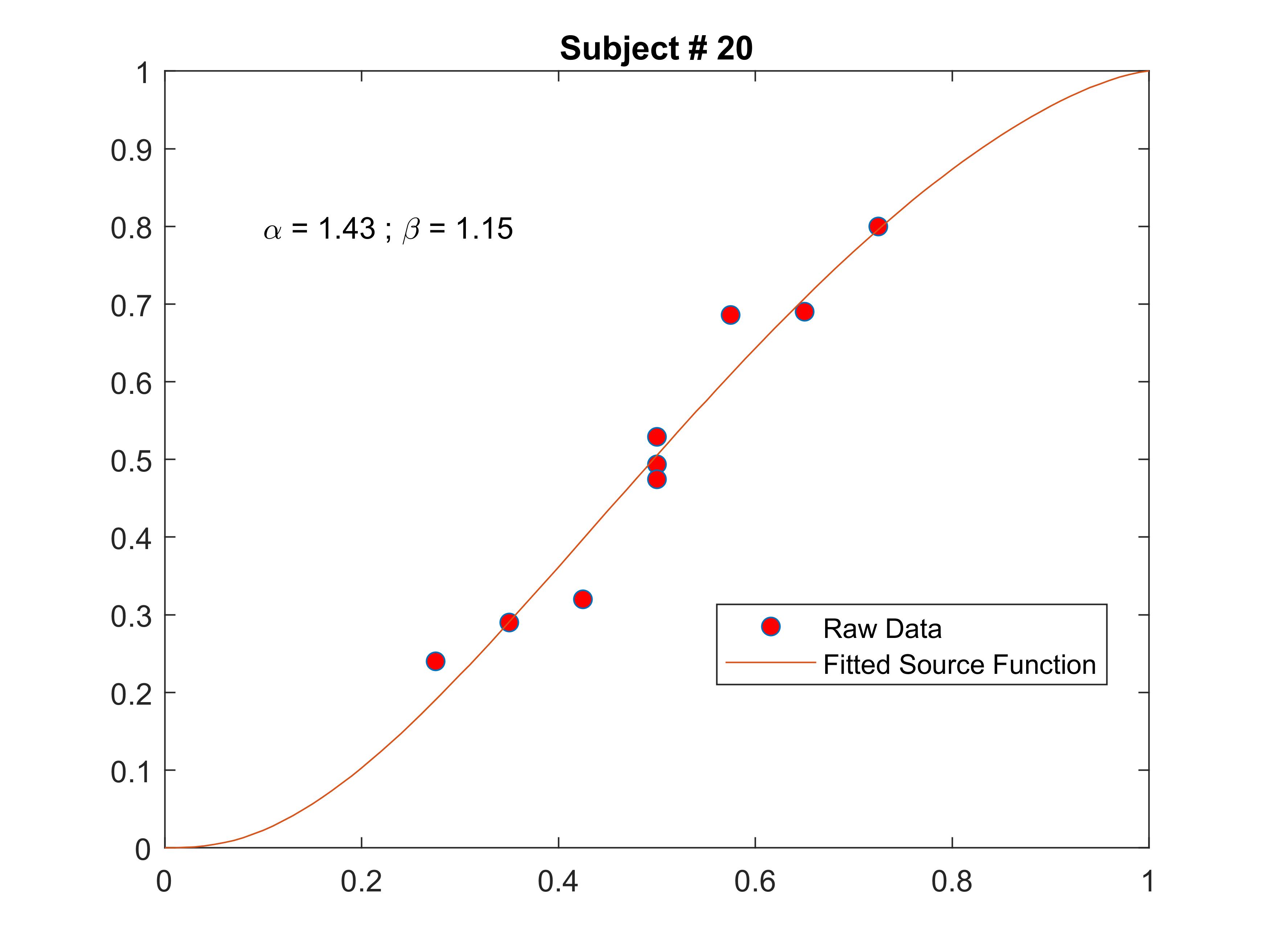 |
| --- | --- |
| 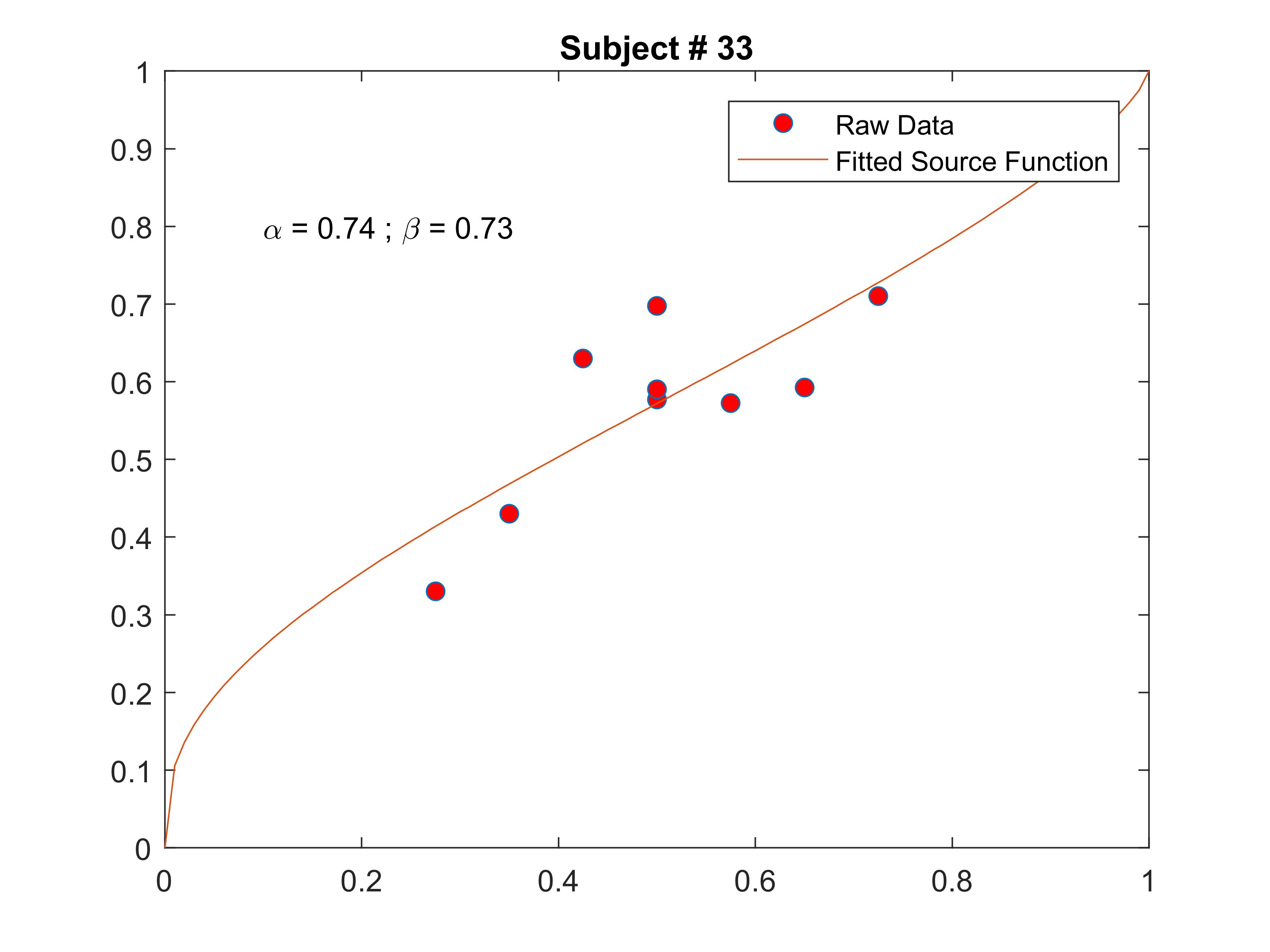 | 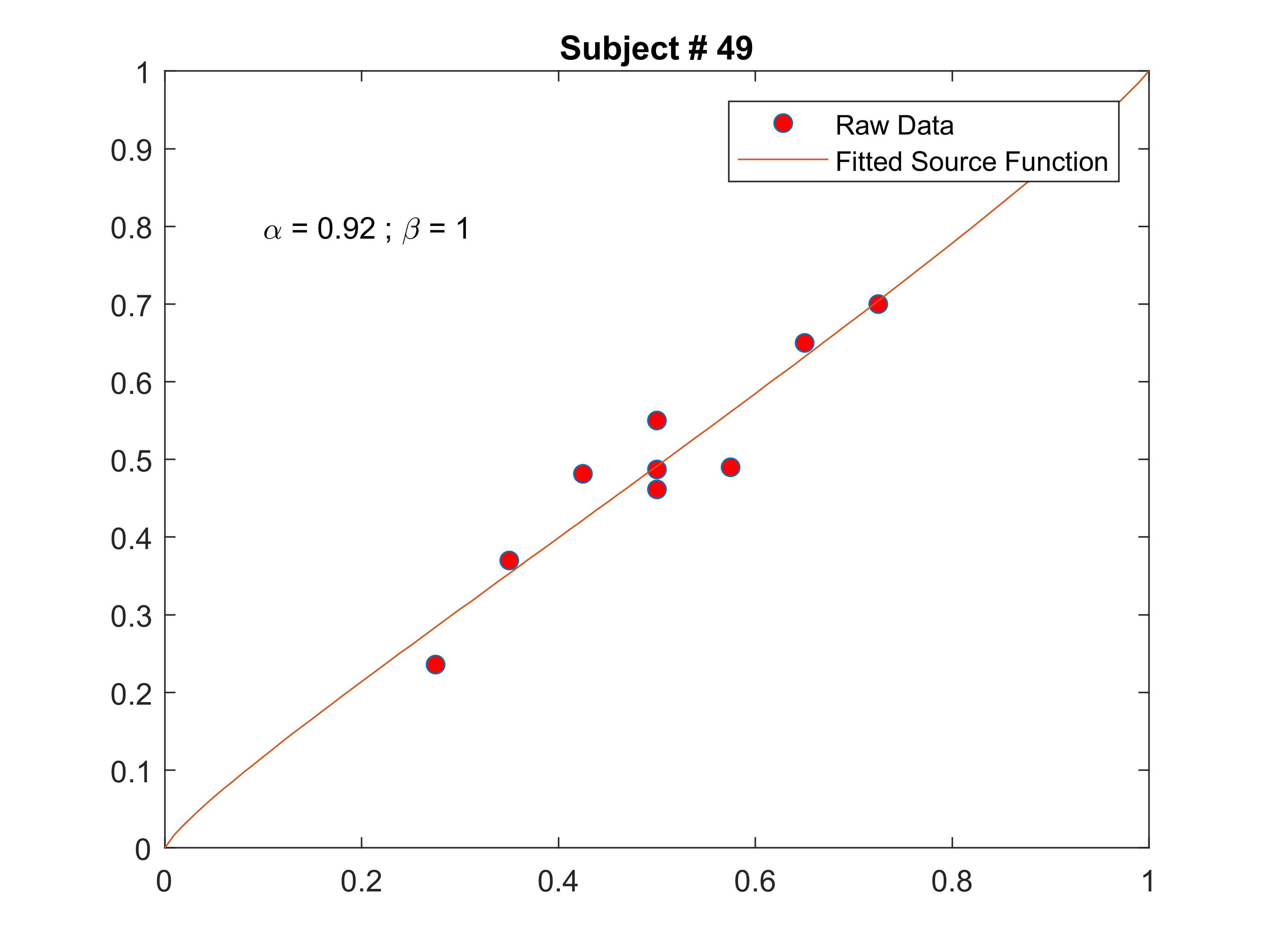 |

Table S4: Ambiguity Attitude (AA) for different strategies

|  | Direct Extrapolation | Reverse Extrapolation | Ignore the information | Variance Maximization |
| --- | --- | --- | --- | --- |
| AA(KWR) | KWR | 1-KWR | 1/2 | No explicit formula |

Variance Maximization does not explicitly propose a specific function for AA. Instead, it regulates the unknown component in a manner that consistently maintains the weighting function at 0.5.
