# Supplementary figures and images for: Uncertain choices with asymmetric information: how clear evidence and ambiguity interact?
Source: Front Psychol. 2024 Dec 19;15:1509320. doi: 10.3389/fpsyg.2024.1509320 (PMC11696535; doi:10.3389/fpsyg.2024.1509320)

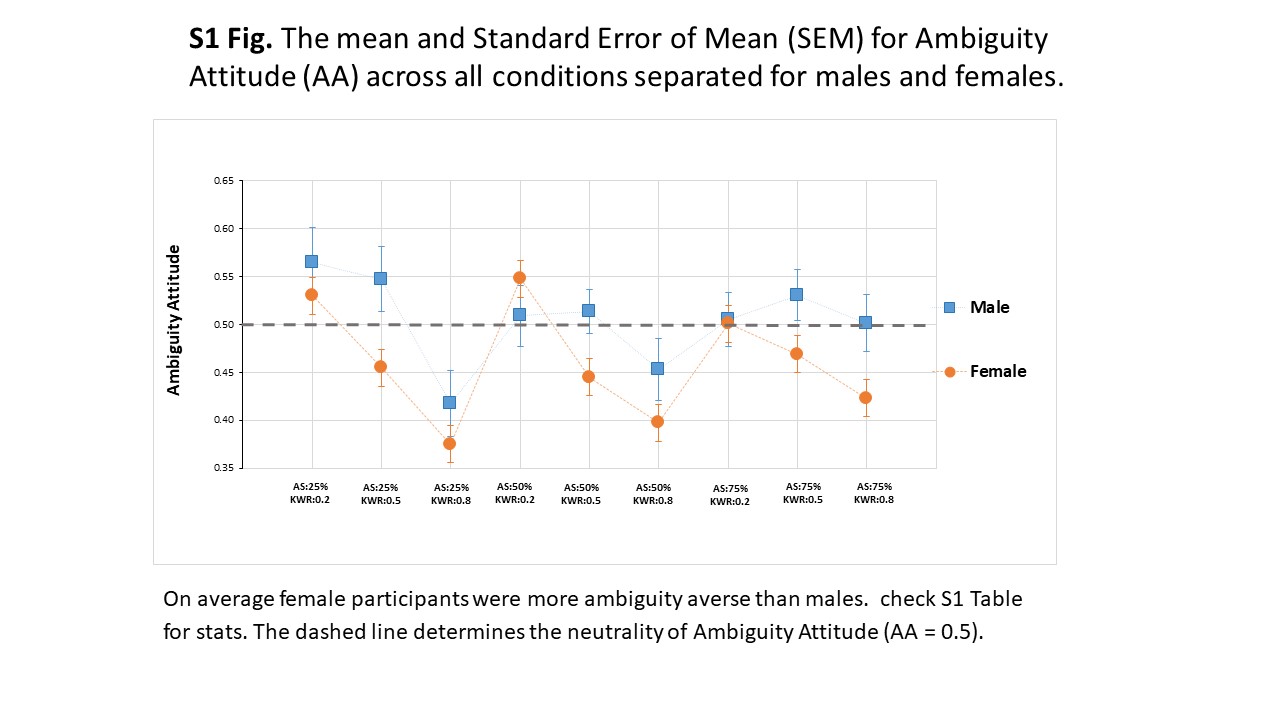

Supplement: Supplementary file 2 [file Image_1.JPEG]

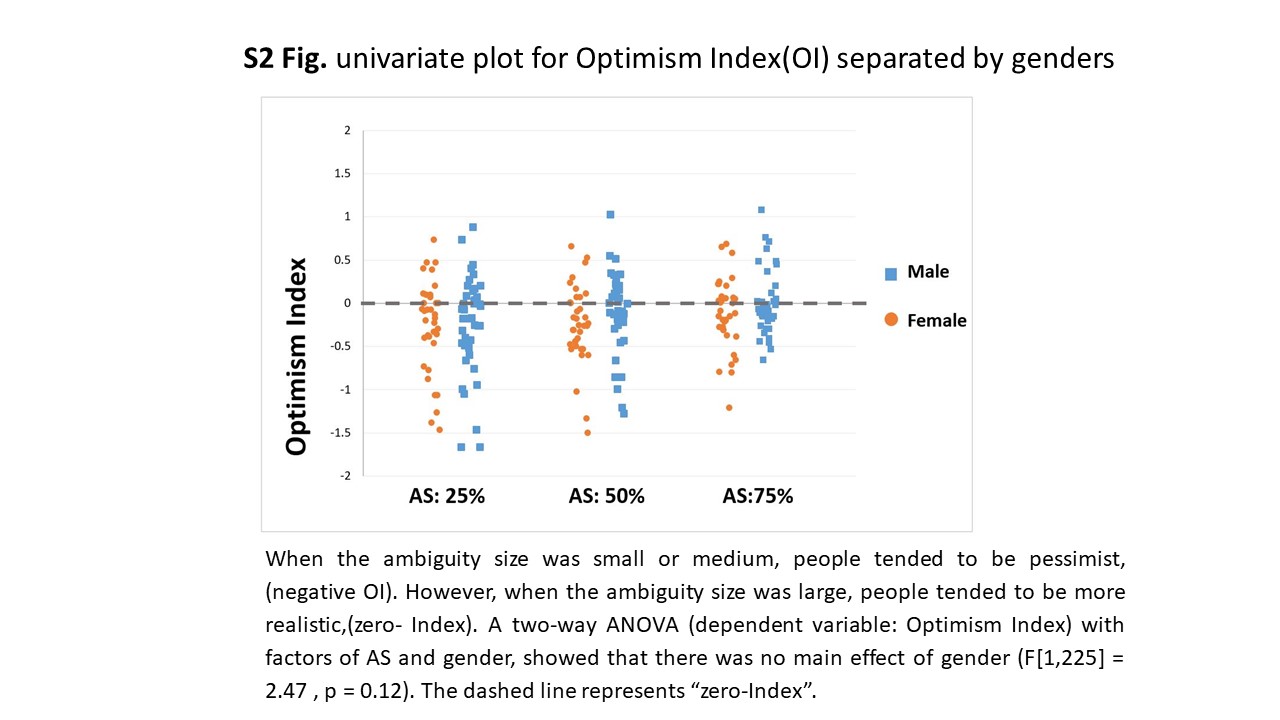

Supplement: Supplementary file 3 [file Image_2.JPEG]

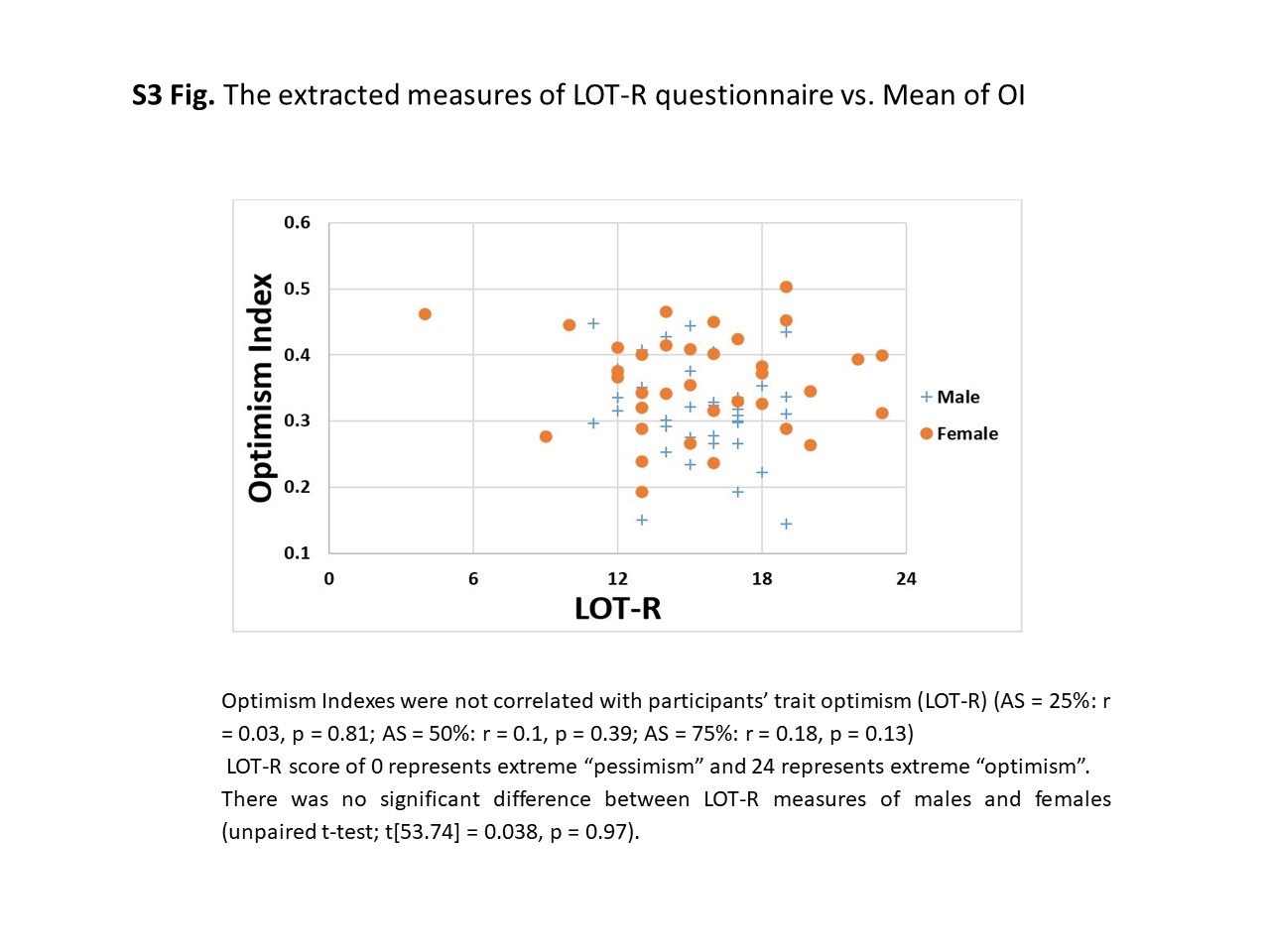

Supplement: Supplementary file 4 [file Image_3.JPEG]
