# Supplementary material for: Uncertain choices with asymmetric information: how clear evidence and ambiguity interact?
Source: Front Psychol. 2024 Dec 19;15:1509320. doi: 10.3389/fpsyg.2024.1509320 (PMC11696535; doi:10.3389/fpsyg.2024.1509320)
Supplement: Supplementary file 5 [file Table_1.docx]

**S1 Table. Results of 3-way ANOVA analysis on Ambiguity Attitude, X1: Ambiguity Size, X2: Known Winning Ratio, X3: Gender**

| **Source** | **Sum Sq.** | **d.f.** | **Mean Sq.** | **F** | **Prob. >F** |
| --- | --- | --- | --- | --- | --- |
| **Ambiguity Size** | 0.01 | 2 | 0.01 | 0.14 | 0.87 |
| **Known Winning Ratio** | 1.14 | 2 | 0.57 | 12.18 | 6.3E-6*** |
| **Gender** | 0.34 | 1 | 0.34 | 7.24 | 0.01* |
| **X1*X2** | 0.26 | 4 | 0.07 | 1.40 | 0.23 |
| **X1*X3** | 0.02 | 2 | 0.01 | 0.25 | 0.78 |
| **X2*X3** | 0.18 | 2 | 0.09 | 1.88 | 0.15 |
| **Error** | 31.92 | 679 | 0.05 | [] | [] |
| **Total** | 33.86 | 692 | [] | [] | [] |

Significance level, * *p* < 0.05, ** *p* < 0.01, *** *p* < 0.001

A 3-way mixed ANOVA (KWR: negative, neutral and positive, AS: small, medium and large, Gender: male and female, AA: dependent variable) was employed showing a significant main effect of KWR [F(2,679) = 12.18, p = 6.3e-6]. There was also a significant effect of gender [F(1,679) = 7.24, p = 0.01], but no main effect of ambiguity size [F[2,679] = 0.14, p = 0.87] and no significant interaction between the independent variables.
