# Supplementary material for: Uncertain choices with asymmetric information: how clear evidence and ambiguity interact?
Source: Front Psychol. 2024 Dec 19;15:1509320. doi: 10.3389/fpsyg.2024.1509320 (PMC11696535; doi:10.3389/fpsyg.2024.1509320)
Supplement: Supplementary file 6 [file Table_2.docx]

**S2 Table. Two-sided t-test analysis on the extracted Optimism Indexes with zero.**

| Ambiguity Size | Gender | Slope | 95% CI | t stat. | P-Value | d.f. |
| --- | --- | --- | --- | --- | --- | --- |
| **25%** | Male | -0.24 | [-0.43, -0.06] | -2.72 | 0.009** | 40 |
|  | Female | -0.26 | [-0.44, -0.08] | -2.90 | 0.006** | 35 |
|  | All | -0.25 | [-0.37, -0.12] | -3.99 | 1.00E-04*** | 76 |
| **50%** | Male | -0.09 | [-0.24, 0.05] | -1.27 | 0.21 | 40 |
|  | Female | -0.25 | [-0.40, -0.10] | -3.29 | 0.002** | 35 |
|  | All | -0.16 | [-0.27, -0.06] | -3.14 | 0.002** | 76 |
| **75%** | Male | -0.01 | [-0.12, 0.11] | -0.1 | 0.92 | 40 |
|  | Female | -0.13 | [-0.27, 0.01] | -1.89 | 0.07 | 35 |
|  | All | -0.06 | [-0.15, 0.02] | -1.42 | 0.16 | 76 |

Significance level, * *p* < 0.05, ** *p* < 0.01, *** *p* < 0.001

In our empirical data, a comparison between Optimism Index(OI) in AS= 75% with zero confirmed that there was no significant difference (one sample t-test with zero; AS = 75%; t[76] = -1.42 , p = 0.16). Moreover, the OIs in small and medium ambiguity size conditions were significantly less than zero (one sample t-test with zero; AS= 50%: t[76] =-3.14, p =0.002; AS= 25%: t[76] = -3.99, p = 1.00E-04)
